# Supplementary material for: UK Dietary Practices for Tyrosinaemias: Time for Change
Source: Nutrients. 2022 Dec 7;14(24):5202. doi: 10.3390/nu14245202 (PMC9787818; doi:10.3390/nu14245202)
Supplement: Supplementary file 1 [file nutrients-14-05202-s001.zip › nutrients-1987389-supplementary-1.pdf]

Supplementary Table S1: Protein, tyrosine and phenylalanine content of fruits and vegetables (0.1 to ≤4g/ 100g per fruit and vegetable) using four data bases measuring: protein (g/100g), tyrosine (mg/100g), phenylalanine (mg/ 100g) and the percentage of tyrosine to phenylalanine and percentage of tyrosine to protein. Table describes the current and proposed exchange system used to count fruits and vegetables using protein or tyrosine/ phenylalanine analysis.

Legend: sources of protein, tyrosine and phenylalanine measurements
NSPKU *National society for phenylketonuria (NSPKU) data base (Weetch 2006)*
Bremmer *Analysis taken from B Bremmner et al 1996*
McCance *McCance and Widdowson's The Composition of Foods*' 1980 First supplementary amino acid mg/100g foods (McCance, Widdowson, Paul, Southgate, Russell, Great Britain Medical Research Council, 4th revised and extended edition);
USA United States of America Department of Agriculture (USDA: United States Department of Agriculture, Agriculture Research Service www.usda.gov);
Mevalia Mevalia web site data base (www. Mavalia.com) based on Frida.fooddata.dk version 4 National Food Institute, Technical University of Denmark.
EF exchange free

Analysis of protein, tyrosine and phenylalanine, and the percentage of tyrosine to phenylalanine and tyrosine to protein for vegetables 0.1 to ≤4g/100g

Source of analysis	Food	Cooked/ uncooked or not stated	Protein / 100g	Tyrosine mg/100g	Phenylalanine mg/100g	% Tyr to Phe (rounded up to nearest whole number)	% Tyr to protein (rounded up to nearest whole number)	Proposed Exchange/exchange free	Exchange system based on protein	Exchange system based on Phe/Tyr
**Vegetables containing protein <0.1 to 1.0g/100g**										
USA	Butternut Squash	Raw	1.0	34	39	87	3	EF	EF	EF
USA	Butternut Squash	Baked	0.9	30	35	86	3	EF	EF	EF
USA	Acorn Squash	Raw	0.8	27	31	87	3	EF	EF	EF
USA	Acorn Squash	Baked	1.1	38	44	86	3	EF	EF	EF
USA	Summer Squash	Raw	1.4	35	46	76	3	EF	EF	EF
USA	Summer Squash	Cooked	0.9	24	32	75	3	EF	EF	EF
USA	Winter Squash	Raw	1.0	49	57	86	5	EF	EF	EF
USA	Winter Squash	Cooked	0.9	30	35	86	3	EF	EF	EF
USA	Spaghetti Squash	Raw	0.6	20	24	83	3	EF	EF	EF
USA	Spaghetti Squash	Cooked	0.7	20	24	83	3	EF	EF	EF
Mevalia	Squash all varieties	Raw	1.4	36	47	77	3	EF	EF	EF
Bremmer	Squash	Raw	0.9	31	52	60	3	EF	EF	EF
Bremmer	Carrots	Raw fresh	0.5	12	18	67	2	EF	EF	EF

Source of analysis	Food	Cooked/ uncooked or not stated	Protein / 100g	Tyrosine mg/100g	Phenylalanine mg/100g	% Tyr to Phe (rounded up to nearest whole number)	% Tyr to protein (rounded up to nearest whole number)	Proposed Exchange/exchange free	Exchange system based on protein	Exchange system based on Phe/Tyr
USA	Carrots	Raw frozen	0.9	28	37	76	3	EF	EF	EF
Mevalia	Carrots	Raw Frozen	0.9	24	39	62	3	EF	EF	EF
Bremmer	Cabbage red	Raw	0.8	18	27	67	2	EF	EF	EF
McCance	Cabbage red	Unstated	0.8	32	51	63	4	EF	EF	EF
Bremmer	Cabbage red	Jar	0.8	12	24	50	2	EF	EF	EF
USA	Cabbage red	Raw	1.4	22	36	61	2	EF	EF	EF
Mevalia	Cabbage red	Raw	1.5	17	27	63	1	EF	EF	EF
NSPKU	Chayote	Raw	0.8	16	18	89	2	EF	EF	EF
USA	Cucumber	Raw	0.7	11	19	58	2	EF	EF	EF
Bremmer	Cucumber	Raw	1.0	6	12	50	1	EF	EF	EF
Mevalia	Cucumber	Raw	0.7	10	17	59	1	EF	EF	EF
Bremmer	Olives green	Jar	0.9	22	29	76	2	EF	EF	EF
Bremmer	Olives	Jar	0.9	24	34	71	3	EF	EF	EF
Bremmer	Oives black	Jar	0.9	25	37	68	3	EF	EF	EF
Mevalia	Pepper green	Raw	0.9	13	27	48	1	EF	EF	EF
USA	Peppers red	Raw	1.0	9	50	18	1	EF	EF	EF
Mevalia	Peppers red	Raw	1.3	19	40	48	1	EF	EF	EF
USA	Tomatoes	Raw	0.9	14	27	52	2	EF	EF	EF
Mevalia	Tomatoes	Raw	0.7	27	20	135	4	EF	EF	EF
USA	Tomato puree	Raw	1.7	22	35	63	1	EF	EF	EF
USA	Water chestnuts	Raw	0.9	10	30	33	1	EF	EF	EF
**Vegetables containing protein 1.1 to 2.0g/100g**										
NSPKU	Baby corn	Cooked	2.0	71	78	91	4	EF	EF	EF
NSPKU	Baby corn	Cooked	2.0	52	61	85	3	EF	EF	EF
USA	Bok Choy	Raw	1.5	29	44	66	2	EF	EF	EF

Source of analysis	Food	Cooked/ uncooked or not stated	Protein / 100g	Tyrosine mg/100g	Phenylalanine mg/100g	% Tyr to Phe (rounded up to nearest whole number)	% Tyr to protein (rounded up to nearest whole number)	Proposed Exchange/exchange free	Exchange system based on protein	Exchange system based on Phe/Tyr
Mevalia	Pak Choi	Raw	1.5	29	43	67	2	EF	EF	EF
Bremmer	Beetroot	Raw	1.6	16	13	123	1	EF	EF	EF
McCance	Beetroot	Unstated	1.6	46	46	100	3	EF	EF	EF
USA	Beetroot	Raw	1.6	38	46	83	2	EF	EF	EF
Mevalia	Beetroot	Raw	1.7	14	16	88	1	EF	EF	EF
Mevalia	Beetroot	Canned	1.0	8	9	89	1	EF	EF	EF
USA/GMDI	Breadfruit	Raw	1.1	19	26	73	2	EF	EF	EF
USA	Cauliflower	Cooked	1.8	40	66	61	2	EF	EF	EF
USA	Cauliflower	Raw	1.9	51	65	78	3	EF	EF	EF
USA	Cauliflower	Cooked	1.6	35	58	60	2	EF	EF	EF
McCance	Cauliflower	Unstated	1.8	27	63	43	2	EF	EF	EF
Bremmer	Cauliflower	Raw	1.8	41	82	50	2	EF	EF	EF
Mevalia	Cauliflower	Raw	2.4	49	80	61	2	EF	EF	EF
Mevalia	Cauliflower	Frozen	2.0	45	71	63	2	EF	EF	EF
USA	Courgette/ Zucchini	Raw	1.2	32	43	74	3	EF	EF	EF
USA	Courgette/ Zucchini	Cooked	1.1	17	22	77	2	EF	EF	EF
Mevalia	Courgette/ Zucchini	Raw	1.2	31	43	72	3	EF	EF	EF
Mevalia	Cabbage white	Raw	1.2	12	22	55	1	EF	EF	EF
USA	Cabbage	Raw	1.3	19	32	59	1	EF	EF	EF
USA	Cassava	Raw	1.4	17	26	65	1	EF	EF	EF
NSPKU	Dudhi	Cooked	1.8	12	17	71	1	EF	EF	EF
NSPKU	Eddoes	Raw	1.5	58	71	82	4	EF	EF	EF

Source of analysis	Food	Cooked/ uncooked or not stated	Protein / 100g	Tyrosine mg/100g	Phenylalanine mg/100g	% Tyr to Phe (rounded up to nearest whole number)	% Tyr to protein (rounded up to nearest whole number)	Proposed Exchange/exchange free	Exchange system based on protein	Exchange system based on Phe/Tyr
USA/GMDI	Jackfruit	Raw	1.7	103	52	198	6	EF	EF	EF
Bremmer	Kohlrabi	Raw	1.7	7	21	33	1	EF	EF	EF
Bremmer	Lettuce	Raw	1.2	11	37	30	1	EF	EF	EF
Bremmer	Lettuce iceberg	Raw	1.2	15	33	45	1	EF	EF	EF
Mevalia	Lettuce iceberg	Raw	1.0	8	26	31	1	EF	EF	EF
Bremmer	Lettuce lambs	Raw	1.2	51	95	54	4	EF	EF	EF
USA	Lettuce cos/romaine	Raw	1.2	25	65	38	2	EF	EF	EF
Mevalia	Lettuce cos/ romaine	Raw	1.2	26	65	40	2	EF	EF	EF
Bremmer	Leeks	Raw	1.6	16	37	43	1	EF	EF	EF
USA	Leeks	Raw	1.5	41	55	75	3	EF	EF	EF
USA	Leeks	Cooked	0.8	22	30	73	3	EF	EF	EF
Mevalia	Leek	Raw	1.9	29	50	58	2	EF	EF	EF
NSPKU	Lotus Roots§	Cooked	1.9	56	63	89	3	EF	EF	EF
USA	Lotus Root	Cooked boiled	1.6	17	28	61	1	EF	EF	EF
USA	Lotus Root	Raw	2.6	29	47	61	1	EF	EF	EF
NSPKU	Okra	Cooked	2.8	38	64	59	1	EF	EF	EF
USA	Okra	Cooked	1.9	81	61	132	4	EF	EF	EF
Mevalia	Okra	Raw	2.0	86	64	134	4	EF	EF	EF
Mevalia	Parsnips	Raw	2.1	33	64	52	2	EF	EF	EF
USA	Parsnip	Cooked	1.3	20	30	67	2	EF	EF	EF
USA	Parsnip	Raw	1.2	18	27	67	2	EF	EF	EF
Bremmer	Potatoes	Cooked boiled	1.8	24	80	30	1	Exchange	56g	80g
McCance	Potatoes	Cooked boiled	1.8	44	62	71	2	Exchange	56g	80g
USA	Potatoes	Cooked boiled	1.9	69	83	83	4	Exchange	53g	80g
Mevalia	Potatoes	Raw	1.9	37	77	48	2	Exchange	53g	80g

Source of analysis	Food	Cooked/ uncooked or not stated	Protein / 100g	Tyrosine mg/100g	Phenylalanine mg/100g	% Tyr to Phe (rounded up to nearest whole number)	% Tyr to protein (rounded up to nearest whole number)	Proposed Exchange/exchange free	Exchange system based on protein	Exchange system based on Phe/Tyr
Mevalia	Potatoes	Canned	1.4	52	63	83	4	Exchange	71g	100g
Bremmer	Potatoes jacket	Cooked with skin	1.9	43	74	57	2	Exchange	53g	80g
USA	Potato jacket	Cooked with skin	2.1	49	83	59	2	Exchange	48g	80g
NSPKU	Potato purple[§]	Cooked	1.7	5	54	98	3	Exchange	59g	80g
NSPKU	Runner beans	Cooked	1.6	43	61	70	3	EF	EF	EF
Bremmer	Runner beans	Raw	1.6	33	48	69	2	EF	EF	EF
USA	Runner beans	Cooked	1.5	34	54	63	2	EF	EF	EF
NSPKU	Samphire	Cooked	1.2	32	51	63	3	EF	EF	EF
USA	Savoy Cabbage	Raw	2.0	34	64	53	2	EF	EF	EF
Bremmer	Savoy Cabbage	Raw		38	70	54		EF	EF	EF
Mevalia	Savoy Cabbage	Raw	2.0	35	64	55	2	EF	EF	EF
NSPKU	Savoy Cabbage	Cooked	1.5	43	52	83	3	EF	EF	EF
USA	Savoy Cabbage	Cooked	1.8	31	58	53	2	EF	EF	EF
USA	Sweet potato	Frozen uncooked	1.7	70	102	69	4	EF	EF	EF
USA	Sweet potatoes	Raw	1.6,	34	89	38	2	EF	EF	EF
USA	Sweet potatoes	Cooked	1.4	68	99	70	5	EF	EF	EF
USA	Sweet potatoes	Cooked, boiled without skin	1.4	30	78	39	2			
McCance	Sweet potatoes	Unstated	1.2	41	89	38	3	EF	EF	EF
Mevalia	Sweet potato	Raw	1.3	29	73	40	2	EF	EF	EF
USA	Yam	Raw	1.5	40	71	62	3	EF	EF	EF
USA	Yam	Boiled	1.5	39	69	57	3	EF	EF	EF
Mevalia	Yam	Raw	1.5	39	71	55	3	EF	EF	EF
**Vegetables containing protein 2.1 to 3.0g /100g**										
Mevalia	Asparagus	Canned	1.6	36	44	82	2	Exchange	63g	60g
Mevalia	Asparagus	Raw	1.8	49	40	123	3	Exchange	56g	60g
USA	Asparagus	Raw	2.2	52	75	69	2	Exchange	45g	60g
USA	Asparagus	Cooked	2.2	52	75	69	2	Exchange	45g	60g
USA	Asparagus	Cooked	2.4	57	82	70	1	Exchange	42g	60g
McCance	Asparagus	Unstated	2.4	70	86	81	3	Exchange	42g	60g

Source of analysis	Food	Cooked/ uncooked or not stated	Protein / 100g	Tyrosine mg/100g	Phenylalanine mg/100g	% Tyr to Phe (rounded up to nearest whole number)	% Tyr to protein (rounded up to nearest whole number)	Proposed Exchange/exchange free	Exchange system based on protein	Exchange system based on Phe/Tyr
Mevalia	Bean sprouts	Raw	3.0	53	120	44	2	Exchange	33g	60g
Bremmer	Broccoli	Raw	2.0	62	145	43	3	Exchange	50g	60g
USA	Broccoli	Raw	2.6	50	118	42	2	Exchange	38g	60g
USA	Broccoli	Cooked	2.4	60	116	52	3	Exchange	42g	60g
NSPKU	Choi Sum	Raw	3.0	77	139	55	3	Exchange	33g	35g
NSPKU	Hearts of palm	Cooked	2.4	63	83	76	3	Exchange	42g	60g
USA	Hearts of palm	Canned	2.5	49	98	50	2	Exchange	40g	60g
Mevalia	Kale	Frozen	2.1	57	84	68	3	Exchange	48g	35g
Mevalia	Kale	Canned	2.2	74	110	67	3	Exchange	45g	35g
Mevalia	Kale	Raw	4.7	110	200	55	2	Exchange	21g	35g
Bremmer	Kale	Raw frozen	3.4	56	106	53	2	Exchange	29g	35g
USA	Kale	Raw	2.9	103	150	69	4	Exchange	34g	35g
USA	Kale	cooked	2.9	104	150	69	4	Exchange	34g	35g
NSPKU	Kale curly	Cooked	3.4	92	150	61	3	Exchange	29g	35g
Bremmer	Mushrooms	Raw	2.5	42	75	56	2	EF	40g	EF
Mevalia	Mushrooms	Raw	2.9	46	70	66	2	EF	34g	EF
Mevalia	Mushrooms	Canned	2.5	40	60	67	2	EF	40g	EF
USA	Mushroom's shitake		1.6	44	67			EF	63g	EF
USA	Mushrooms white button	Raw	3.1	44	85	52	1	EF	32g	EF
USA	Mushrooms white button	Cooked boiled	2.2	31	60	52	1	EF	45g	EF
USA	Mushrooms	Canned, drained	1.9	26	52	50	1	EF	53g	EF
NSPKU	Romanesco	Cooked	3.0	94	149	63	3	Exchange	33g	35g
USA	Sweet corn	Cooked boiled	3.0	112	138	81	4	Exchange	33g	35g

Source of analysis	Food	Cooked/ uncooked or not stated	Protein / 100g	Tyrosine mg/100g	Phenylalanine mg/100g	% Tyr to Phe (rounded up to nearest whole number)	% Tyr to protein (rounded up to nearest whole number)	Proposed Exchange/exchange free	Exchange system based on protein	Exchange system based on Phe/Tyr
USA	Spinach	Raw	2.9	108	129	84	4	Exchange	34g	25g
USA	Spinach	Raw	2.9	108	129	84	4	Exchange	34g	25g
Bremmer	Spinach	Frozen	2.1	64	99	65	3	Exchange	48g	25g
Bremmer	Spinach	Raw	2.6	47	62	76	2	Exchange	38g	25g
Mevalia	Spinach	Frozen	2.1	50	97	52	2	Exchange	48g	25g
Mevalia	Spinach	Raw	2.6	63	120	53	2	Exchange	38g	25g
NSPKU	Vine leaves	Cooked	3.0	119	178	67	3	Exchange	33g	30g
USA	Watercress	Raw	2.3	63	114	55	3	EF	43g	EF
NSPKU	Watercress	Raw	3.0	17	26	65	1	EF	33g	EF
Mevalia	Watercress	Raw	1.7	46	84	55	3	EF	59g	EF
USA	Whole hearts of palm	Canned	2.5	49	98	50	2	Exchange	40g	60g
NSPKU	Whole hearts of palm	Cooked	2.4	63	83	76	3	Exchange	42g	60g
**Vegetables containing protein <4.0g/100g**										
USA	Broad beans	Cooked	4.8	168	195	86	4	Exchange	21g	20g
Bremmer	Brussels sprouts	Raw	3.5	38	77	49	1	Exchange	29g	60g
Mevalia	Brussels sprouts	Raw	4.5	73	130	56	2	Exchange	22g	60g
Mevalia	Brussels sprouts	Frozen	3.3	53	95	56	2	Exchange	30g	60g
NSPKU	Chestnuts	Canned	3.1	72	137	53	2	Exchange	32g	40g
USA	Chestnuts	Roasted	3.2	88	134	66	3	Exchange	31g	40g
USA	Chestnuts	Boiled	2.0	55	84	65	3	Exchange	50g	40g
Mevalia	Chestnuts	Raw	2.0	54	83	65	3	Exchange	50g	40g
NSPKU	Kalettes	Cooked	3.5	94	127	74	3	Exchange	29g	40g
Bremmer	Potato Chips	Fried	3.5	80	119	67	2	Exchange	29g	45g
McCance	Potato Chips	Unstated	3.5	120	170	71	3	Exchange	29g	45g
USA	Potato French fries	Fried	2.5	85	107	79	3	Exchange	40g	45g

Source of analysis	Food	Cooked/ uncooked or not stated	Protein / 100g	Tyrosine mg/100g	Phenylalanine mg/100g	% Tyr to Phe (rounded up to nearest whole number)	% Tyr to protein (rounded up to nearest whole number)	Proposed Exchange/exchange free	Exchange system based on protein	Exchange system based on Phe/Tyr
Mevalia	Potato chips	Fried	3.0	94	200	47	3	Exchange	33g	45g
NSPKU	Pea shoots	Raw	3.1	156	271	58	5	Exchange	32g	20g
Bremmer	Peas	Canned	4.3	115	291	40	3	Exchange	23g	25g
Bremmer	Peas	Frozen	4.3	87	194	45	2	Exchange	23g	25g
USA	Peas	Raw	4.3	167	201	83	4	Exchange	23g	25g
USA	Peas	Cooked	5.4	112	198	57	2	Exchange	19g	25g
Mevalia	Peas	Canned	5.9	170	250	68	3	Exchange	17g	25g
Mevalia	Peas	Raw	5.9	130	210	62	2	Exchange	17g	25g
NSPKU	Mange tout	Cooked	3.6	59	93	63	3	Exchange	28g	60g
USA	Sugar snap/snow peas	Raw	2.8	99	90	110	4	Exchange	36g	25g
USA	Sugar snap/snow peas	Cooked	3.3	115	105	110	3	Exchange	36g	25g
NSPKU	Sugar snap peas	Cooked	3.4	55	88	63	2	Exchange	29g	60g
NSPKU	Sugar snap peas	Cooked	3.4	47	75	63	1	Exchange	29g	60g
NSPKU	Rocket	Raw	3.6	61	143	43	2	Exchange	28g	35g

Analysis of protein, tyrosine and phenylalanine, and the percentage of tyrosine to phenylalanine and tyrosine to protein for fruits 0.1 to ≤4g/100g

Source of analysis	Food	Cooked/ uncooked or not stated	Protein / 100g	Tyrosine mg/ 100g	Phenylalanine mg/ 100g	% Tyr to Phe (rounded up to nearest whole number)	% Tyr to protein (rounded up to nearest whole number)	Proposed Exchange/exchange free	Exchange system based on protein	Exchange system based on Phe/Tyr
**Fruits containing protein 0.1 to 1.0g/100g**										
Bremmer	Apple Granny Smith	Raw	0.6	4	8	50	1	EF	EF	EF
Bremmer	Apple Golden Delicious	Raw	0.3	4	8	50	1	EF	EF	EF
Bremmer	Apple Cox Orange	Raw	0.3	4	8	50	1	EF	EF	EF
USA	Apples Golden Delicious	Raw	0.3	1	7	14	0.5	EF	EF	EF
USA	Apples generic	Raw	0.3	1	8	13	0.5	EF	EF	EF
Mevalia	Apples generic	Raw	0.3	4	7	57	1	EF	EF	EF
Bremmer	Grapes	Raw	0.7	5	16	31	1	EF	EF	EF
USA	Grapes	Raw	0.6	11	13	85	2	EF	EF	EF
Mevalia	Grapes	Raw	0.5	9	10	90	2	EF	EF	EF
Mevalia	Figs	Raw	0.8	32	18	178	4,	EF	EF	EF
USA	Figs	Dried	3.3	41	76	54	1	EF	EF	EF
USA	Figs	Raw	0.5	20	12	166	4	EF	EF	EF
Bremmer	Melon honeydew	Raw	0.6	3	14	21	0.5	EF	EF	EF
USA	Melon honeydew	Raw	0.5	10	15	67	2	EF	EF	EF
Mevalia	Melon honeydew	Raw	0.6	10	16	63	2	EF	EF	EF
Bremmer	Melon water	Raw	0.5	3	21	14	1	EF	EF	EF
USA	Melon water	Raw	0.6	12	15	80	2	EF	EF	EF
Mevali	Melon water	Raw	0.8	15	19	79	2	EF	EF	EF
USA	Melon Cantaloupe	Raw	0.8	11	19	58	1	EF	EF	EF
Mevalia	Melon Cantaloupe	Raw	0.8	13	23	56	2	EF	EF	EF

USA	Oranges	Raw	0.9	16	31	52	2	EF	EF	EF
Mevalia	Oranges	Raw	0.9	13	21	62	1	EF	EF	EF
USA	Pears	Raw	0.4	2	13	15	0.5	EF	EF	EF
Mevalia	Pears	Raw	0.3	5	13	38	2	EF	EF	EF
Mevalia	Pears	Canned	0.3	5	12	42	2	EF	EF	EF
Bremmer	Peach	Raw	1.0	6	10	60	1	EF	EF	EF
USA	Peach	Raw	0.9	14	18	78	2	EF	EF	EF
Bremmer	Pineapple	Raw	0.4	8	12	67	2	EF	EF	EF
NSPKU	Pineapple	Raw	0.5	19	21	90	4	EF	EF	EF
USA	Pineapple	Raw	0.5	19	21	90	4	EF	EF	EF
Mevalia	Pineapple	Raw	0.5	13	13	100	3	EF	EF	EF
Bremmer	Plums	Raw unpeeled stoned	0.6	4	8	50	1	EF	EF	EF
USA	Plums	Raw	0.7	8	14	57	1	EF	EF	EF
Mevalia	Plums	Raw	0.5	9	16	56	2	EF	EF	EF
Bremmer	Strawberry	Raw	0.6	8	20	40	1.5	EF	EF	EF
USA	Strawberry	Raw	0.7	22	19	116	3	EF	EF	EF
Mevalia	Strawberry	Raw	0.7	11	23	48	2	EF	EF	EF
**Fruits containing protein 1.1 to 2.0g /100g**										
NSPKU	Avocado	Raw	1.9	19	34	56	1,	EF	EF	EF
NSPKU	Avocado	Raw	1.9	33	46	72	2	EF	EF	EF
NSPKU	Avocado	Raw	1.9	34	50	68	2	EF	EF	EF
Mevalia	Avocado	Raw	1.9	43	67	64	2	EF	EF	EF
USA	Avocado	Raw	2.0	49	97	51	2	EF	EF	EF
USA	Avocado	Raw	2.0	48	95	51	2	EF	EF	EF
Bremmer	Banana	Raw	1.2	11	28	39	1	EF	EF	EF
USA	Banana	Raw	1.1	9	49	18	1	EF	EF	EF
Mevalia	Banana	Raw	1.3	23	40	58	2	EF	EF	EF
USA	Cherries no stone	Raw	1.1	14	24	58	1	EF	EF	EF
Mevalia	Cherries	Raw	1.3	15	24	58	1	EF	EF	EF
NSPKU	Dragon fruit	Raw	1.2	28	36	78	2	EF	EF	EF

Bremmer	Kiwi	Raw peeled	1.1	16	35	46	1	EF	EF	EF
USA	Kiwi	Raw peeled	2.1	61	79	77	3	EF	EF	EF
Mevalia	Kiwi	Raw	1.0	29	36	81	3	EF	EF	EF
NSPKU	Mulberries	Raw	1.4	34	47	72	2	EF	EF	EF
USA	Nectarines	Raw	1.4	6	9	67	0.5	EF	EF	EF
Mevalia	Nectarines	Raw	1.1	7	11	64	1	EF	EF	EF
**Fruits containing protein >2.0g/100g**										
NSPKU	Passion Fruit	Raw	2.6	39	122	32	2	Exchange	38g	40g
Mevalia	Prune	Dried	2.7	22	51	43	1	EF	37g	EF
